# Metabolomics-Driven Elucidation of Cellular Nitrate Tolerance Reveals Ascorbic Acid Prevents Nitroglycerin-Induced Inactivation of Xanthine Oxidase

**DOI:** 10.3389/fphar.2018.01085

**Published:** 2018-09-25

**Authors:** Elizabeth Rose Axton, Eleonso Cristobal, Jaewoo Choi, Cristobal L. Miranda, Jan Frederik Stevens

**Affiliations:** ^1^The Linus Pauling Institute, Oregon State University, Corvallis, OR, United States; ^2^Department of Pharmaceutical Sciences, Oregon State University, Corvallis, OR, United States; ^3^Department of Environmental and Molecular Toxicology, Oregon State University, Corvallis, OR, United States

**Keywords:** nitrate tolerance, nitroglycerin, xanthine oxidase, ascorbic acid, nitric oxide, metabolomics

## Abstract

Glyceryl trinitrate (GTN) has found widespread use for the treatment of angina pectoris, a pathological condition manifested by chest pain resulting from insufficient blood supply to the heart. Metabolic conversion of GTN, a nitric oxide (NO) pro-drug, into NO induces vasodilation and improves blood flow. Patients develop tolerance to GTN after several weeks of continuous use, limiting the potential for long-term therapy. The mechanistic cause of nitrate tolerance is relatively unknown. We developed a cell culture model of nitrate tolerance that utilizes stable isotopes to measure metabolism of ^15^N_3_-GTN into ^15^N-nitrite. We performed global metabolomics to identify the mechanism of GTN-induced nitrate tolerance and to elucidate the protective role of vitamin C (ascorbic acid). Metabolomics analyses revealed that GTN impaired purine metabolism and depleted intracellular ATP and GTP. GTN inactivated xanthine oxidase (XO), an enzyme that is critical for the metabolic bioactivation of GTN into NO. Ascorbic acid prevented inactivation of XO, resulting in increased NO production from GTN. Our studies suggest that ascorbic acid has the ability to prevent nitrate tolerance by protecting XO, but not aldehyde dehydrogenase (another GTN bioactivating enzyme), from GTN-induced inactivation. Our findings provide a mechanistic explanation for the previously observed beneficial effects of ascorbic acid in nitrate therapy.

## Introduction

Glyceryl trinitrate (GTN), more commonly known as nitroglycerin in the clinic, has been in use since the 1870’s. GTN relieves chest pain caused by insufficient blood flow, a condition known as angina pectoris. GTN is a pro-drug that is enzymatically bioactivated into nitrite (${\mathrm{NO}}_{2}^{–}$) and nitric oxide (NO) in endothelial cells. NO diffuses into smooth muscle cells, inducing vasodilation and improving blood flow to relieve chest pain. GTN, and other organic nitrates, are a recommended course of treatment for patients with stable coronary artery disease who are still symptomatic despite treatment with aspirin, beta-adrenergic receptor blockers, ACE-inhibitors/AT-1 receptor blockers, and statins (Montalescot et al., 2013). Though GTN is well-known to be bioactivated into NO, recent experimental and clinical studies have determined that NO on its own cannot account for all of the vasodilatory effects, suggesting that GTN has additional vasoactive mechanisms (Kleschyov et al., 2003; Núñez et al., 2005).

Long-term GTN therapy causes the development of nitrate tolerance, which is defined as a reduced response to treatment, or the need to increase dosages to maintain the effects, of organic nitrates. Patients develop tolerance to GTN after several weeks of continuous use, limiting its efficacy as a long-term treatment. Nitrate tolerance is currently managed by implementing daily 12-h nitrate-free intervals. Though effective, this strategy leads to increased cardiovascular events during nitrate-free periods, especially in the early morning (Azevedo et al., 2001). Some of the systemic changes that occur from nitrate therapy include desensitization of soluble guanylate cyclase (sGC) (Rapoport et al., 1987), inactivation of endothelial NO synthase (eNOS) (Münzel et al., 2005), neurohormonal activation and intravascular volume expansion (termed *pseudotolerance*) (Parker et al., 1991), and increased vascular superoxide ($O_{2}^{–}$) and peroxynitrite (ONOO^-^) production (Münzel et al., 1995; Hink et al., 2003). Superoxide and peroxynitrite are byproducts of GTN bioactivation, and are correlated with the development of nitrate tolerance (Dikalov et al., 1998). Nitrate tolerance and its implications on human health have previously been reviewed (Münzel et al., 2014).

The efficacy and safety of nitrates could potentially be improved with co-treatment strategies, preventing the need for nitrate-free intervals. Ascorbic acid (vitamin C) is a potent water-soluble antioxidant that also acts as a cofactor for a number of 2-ketoglutarate dependent dioxygenases. Ascorbic acid enhances endothelium-dependent vasodilation and prevents endothelial dysfunction (Levine et al., 1996; Kugiyama et al., 1998). Several clinical studies have shown that co-treatments with ascorbic acid prevent the development of nitrate tolerance (Bassenge and Fink, 1996; Bassenge et al., 1998; Watanabe et al., 1998), but the underlying mechanism of action remains poorly understood. Continuous 3-day transdermal GTN treatment causes a 450% increase in platelet superoxide production in humans, which was reduced to a 60% increase upon ascorbic acid co-treatment (Mcveigh et al., 2002). In addition to its antioxidant and superoxide scavenging properties, ascorbic acid may prevent nitrate tolerance by increasing intracellular glutathione levels (Johnston et al., 1993), increasing intracellular levels of the eNOS cofactor, tetrahydrobiopterin, to maintain eNOS activity (Huang et al., 2000), or by directly bioactivating GTN into nitrite or NO (Millar, 1995).

Nitrate tolerance has long been regarded as a limitation in nitrate efficacy, but it is now believed to correlate with an onset of potentially harmful vascular abnormalities that increase the risk of adverse cardiovascular outcomes. We conducted a global metabolomics study of a cell culture model of nitrate tolerance as a hypothesis-generating approach to elucidate the mechanism by which ascorbic acid functions to prevent nitrate tolerance. Our results demonstrate that xanthine oxidase (XO) and aldehyde dehydrogenase 2 (ALDH2), both of which are key GTN-bioactivating enzymes (Doel et al., 2000; Li et al., 2005), are inactivated by GTN treatment. We found that ascorbic acid protects XO, but not ALDH, from inactivation and that it restored NO production. Considering that GTN appears to irreversibly damage ALDH2, ascorbic acid supplementation could emerge as an important therapeutic strategy to prevent nitrate tolerance by preserving XO activity.

## Materials and Methods

### Chemicals

All solvents and reagents were commercially available and of analytical grade quality unless otherwise specified. Dulbecco’s Modified Eagle Medium (DMEM), fetal bovine serum (FBS), penicillin-streptomycin (PS), 0.25% trypsin-EDTA and Hank’s Buffered Salt Solution (HBSS) were procured from Invitrogen (Carlsbad, CA, United States). T75 flasks, culture dishes (100 mm) and other sterile plastic ware were purchased from Corning (Corning, NY, United States). 2,3-diaminonaphthalene (DAN) was procured from TCI America (Portland, OR, United States). ^15^N_3_-glyceryl trinitrate (^15^N, 98%) in acetonitrile, sodium ^15^N-nitrite (^15^N, 98%), and sodium ^15^N-nitrate (^15^N, 98%) were from Cambridge Isotope Laboratories (Tewksbery, MA, United States). Sodium ascorbate, bovine XO, human recombinant mitochondrial aldehyde dehydrogenase (ALDH2), diphenyleneiodonium chloride (DPI), allopurinol, disulfiram, L-NAME, and GTN were procured from Sigma Aldrich (St. Louis, MO, United States). LC-MS grade water, methanol, and acetonitrile were purchased from J.T. Baker (Center Valley, PA, United States). Formic acid and ammonium bicarbonate were obtained from Fluka (Buchs, Switzerland).

### Cell Culture

**FIGURE 1:**
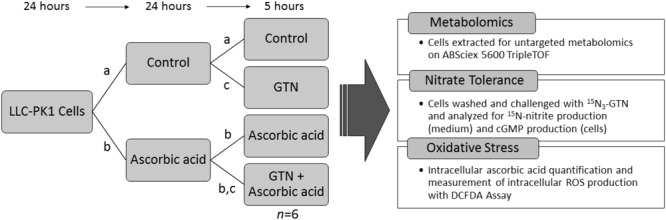
Experimental design. LLC-PK1 cells were plated at a density of 5.0 × 10^5^ cells per 100 mm cell culture dish. 24 h later, they were treated with (a) vehicle control, or (b) 100 μM sodium ascorbate. After 24 h, cells were treated with (a) vehicle control, (b) 100 μM sodium ascorbate, or (c) 1 μM glyceryl trinitrate (GTN). After 5 h, the cells (*n* = 6/group) were analyzed for metabolomics, nitrate tolerance, intracellular ascorbic acid, or intracellular reactive oxygen species (ROS).

Porcine renal epithelial cells (LLC-PK1, ATCC, Manassas, VA, United States) were chosen for metabolomics analysis because of their ability to bioactivate GTN and because they have an intact NO-sGC-cGMP pathway (Hinz and Schröder, 1998b). Cells were maintained under sterile conditions in 75 cm^2^ flasks in DMEM supplemented with 10% FBS and 1% PS, with 5% CO_2_ at 37°C. Cells were used for experiments on passages 5–8 only. LLC-PK1 cells were plated in 100 mm culture dishes at a density of 5.0 × 10^5^ cells in DMEM with 10% FBS and 1% PS. 24 h after plating, cells were treated with 100 μM sodium ascorbate. After a 24-h treatment, the medium was changed and the cells were treated with a second 100 μM dose of sodium ascorbate in DMEM with 1% FBS and 1% PS, and immediately challenged with 1 μM GTN. Treatment groups were (1) vehicle control, (2) GTN, (3) ascorbic acid (ASC), and (4) GTN + ASC. After a 5-h treatment, cells were either extracted for metabolomics analysis or assayed for nitrate tolerance (Nitrite Assay, cGMP Assay), intracellular ascorbic acid, or intracellular reactive oxygen species (ROS) determined by the DCFDA Assay (Experiment Design, **Figure 1**).

We selected this ascorbic acid treatment protocol because it effectively attenuated nitrate tolerance, and it is a physiologically relevant dose. Similarly, the 1 μM GTN dose was chosen because it was previously shown to be effective at inducing nitrate tolerance with no effect on cell viability or proliferation in LLC-PK1 cells (Hinz and Schröder, 1998b; D’souza et al., 2013), while being a sufficient dose for the nitrite method detection limits (Axton et al., 2016). A vehicle control of 2.3 μL acetonitrile was chosen to control for the 2.3 μL of acetonitrile added with the GTN treatment. Cell viability assays demonstrated that neither GTN nor ascorbic acid caused any significant changes in cell viability at the concentrations used for metabolomics analysis, as measured by an MTT Assay (**Supplementary Figure 1**).

### Nitrate Tolerance Analyses

Pre-treated cells (vehicle, GTN, ASC, or GTN + ASC) were washed once with 3 mL warm HBSS. Cells were challenged with 1 μM ^15^N_3_-GTN for 30 min. 100 μL of the medium was transferred to 1.5 mL Eppendorf tubes and immediately assayed for ^15^N-nitrite production.

### Nitrite LC-MS/MS Assay

^15^N-nitrite was measured as a biomarker for bioactivation of ^15^N_3_-GTN. Isotope labeling allowed us to distinguish between endogenous nitrite and nitrite produced from GTN bioactivation. 10 μL of 316 μM 2,3-diaminonaphthalene (DAN) in 0.62 M HCl was added to 100 μL of cell culture medium and incubated at 24°C for 60 min. The reaction was quenched with 5 μL of 2.8 M NaOH. The samples were centrifuged at 16,000 × *g* for 1 min, and the supernatant was transferred to mass spectrometry vials and analyzed for ^15^N-nitrite with the previously described method with minor modifications (Axton et al., 2016). The column was switched to a Poroshell 120 HPH-C18 column (2.7 μm, 2.1 × 50 mm, Agilent, Santa Clara, CA, United States), allowing for a reduced run time of 10 min.

### cGMP ELISA Assay

Cyclic guanosine monophosphate (cGMP) was quantified as a marker for NO production. Pre-treated cells were washed twice with HBSS, then incubated with 0.5 mM isobutylmethylxanthine (IBMX). After addition of GTN to yield a concentration of 1 μM, the cells were incubated for 30 min. Medium was aspirated and the cells were washed twice with HBSS. 5 mL of 0.1 M HCl was added to each cell culture dish and allowed to incubate for 20 min. The cells were scraped and the plate contents were transferred to a 15-mL conical tube and centrifuged at 1,000 × *g* for 10 min. The supernatant was assayed for cGMP according the kit protocol (cGMP ELISA Kit, Cayman Chemical, Ann Arbor, MI, United States). Samples were analyzed on a fluorescence plate reader (Molecular Devices SpectraMax GeminiXS).

### Quantification of Intracellular Ascorbic Acid With HPLC/ECD

Ascorbic acid was quantified with paired ion reverse phase HPLC coupled with electrochemical detection (ECD), as previously described (Frei et al., 1989). In short, cells were harvested with trypsin, and the cell pellet was extracted with 10% perchloric acid with 1 mM diethylenetriamine pentaacetic acid (DTPA). The supernatant was diluted 10× with mobile phase and adjusted to pH 5.0 with 2.58 M K_2_PO_4_ (pH 9.8). Samples were analyzed with a Waters 2695 HPLC equipped with a Supelcosil LC-8 column (25 cm × 4.6 mm i.d., Supelco, St. Louis, MO, United States) and a LC-18 guard column (2 cm × 4.6 mm i.d., Supelco). The eluant (40 mM sodium acetate, 7.5% vol/vol methanol, 0.54 mM DTPA, 1.5 mM dodecyl-triethylammonium phosphate in purified Milli-Q water, taken to pH 4.75 with glacial acetic acid) was delivered at 1.0 mL/min. Analysis was performed on a LC 4B amperometric electrochemical detector equipped with a glassy-carbon working electrode and a Ag/AgCl reference electrode (Bioanalytical Systems, West Lafayette, IN, United States). Ascorbic acid was analyzed with an applied potential of +0.5 V with a sensitivity of 50 nA, and eluted as a single peak at 5.8 min.

### Quantification of Intracellular Reactive Oxygen Species (ROS)

Intracellular production of ROS was quantified with a 2′-7′-dichlorofluorescein diacetate (DCFDA) assay. LLC-PK1 cells were plated in a black 96-well plate (5,000 cells/well). 10 μM H_2_DCFDA (ThermoFisher, Waltham, MA, United States) was added to cells in phenol red-free DMEM with 1% FBS and incubated for 20 min. The DCFDA medium was aspirated and the cells were washed 3× with DMEM. Phenol red-free and FBS-free DMEM was added to the cells. Intracellular production of DCF was measured with a fluorescence spectrophotometer (Ex/Em = 485/530 nm; Molecular Devices SpectraMax GeminiXS) every 20 min for 3 h. Results were processed with SoftMax Pro 5.4.

### Metabolomics Sample Preparation

Treated cells were washed twice with 3 mL of warm HBSS. 3 mL of warm trypsin (0.25%) was added to each cell culture dish and incubated for 3 min. 3 mL of medium was added, and detached cells were removed to 15-mL conical centrifuge tubes. Cells were centrifuged at 4°C at 500 × *g* for 5 min. The liquid was aspirated, and the pellets were washed with cold HBSS 3X. 1 mL of ice-cold degassed methanol: acetonitrile: water (2:2:1) was added to each cell pellet and vortexed for 30 s. The cells were frozen in liquid N_2_ for 60 s, then sonicated and allowed to thaw in a water bath for 10 min. This freeze-thaw cycle was repeated for a total of 3 times. The cell extracts were incubated at -20°C for 1 h, then centrifuged at 4°C at 13,000 × *g* for 15 min. The supernatant was transferred to 1.5 mL Eppendorf tubes and evaporated using a freeze dryer (Labconco FreeZone 6 Plus). The cell extracts were re-suspended in 100 μL ice-cold 50% acetonitrile in water, sonicated at room temperature for 10 min, then centrifuged at 4°C at 13,000 × *g* for 15 min. The supernatant was transferred to mass spectrometry vials (Microsolv, Eatontown, NJ, United States) and frozen at -80°C until analysis.

### Metabolomics Instrumentation

LC-MS/MS based metabolomics was performed as previously described (Kirkwood et al., 2012). In short, high-pressure liquid chromatography (HPLC) was performed on a Shimadzu Nexera system (Shimadzu, Columbia, MD, United States) with a phenyl-3 stationary phase column (Inertsil Phenyl-3, 4.6 × 150 mm, GL Sciences, Torrance, CA, United States) coupled to a quadrupole time-of-flight mass spectrometer (Sciex TripleTOF 5600) operated in information-dependent MS/MS acquisition mode in both positive and negative ion mode. The flow rate was 0.4 ml/min, the injection volume was 10 μL, and the mobile phases consisted of water (A) and methanol (B), both with 0.1% formic acid. The elution gradient was as follows: 0 min, 5% B; 1 min, 5% B; 11 min, 30% B; 23 min, 100% B; 35 min, 100% B; 37 min, 5% B; and 47 min, 5% B. Samples were randomized, and a pooled QC sample was analyzed every 5 samples. Auto-calibration was performed after every two samples.

### Metabolomics Data Processing

All metabolomics samples were analyzed using PeakView with XIC Manager 1.2.0 (Sciex, Framingham, MA, United States) for peak picking, retention time correction, and peak alignment. Metabolite identities were assigned by matching accurate mass (error < 10 ppm), retention time (error < 10%), MS/MS fragmentation (library score > 70), and isotope distribution (error < 20%) with an in-house library consisting of 619 IROA standards (IROA Technology, Bolton, MA, United States) and 30 other commercially available standards, primarily from Sigma–Aldrich (St. Louis, MO, United States) and TCI America (Portland, OR, United States). The peak list was exported to MultiQuant 3.0.2 (ABSciex), and used to create a MultiQuant method. All sample chromatograms were integrated to obtain peak area for all of the assigned metabolites. The results table was exported to MarkerView 1.2.1 (Sciex) to generate log fold-change vs. *p*-value (Volcano) plots.

### Statistical Analysis

All bioassay analyses with 3 or more treatment groups were analyzed with a one-way ANOVA with Bonferroni *post hoc* analysis with a *p*-value of <0.05 indicating significance. Values of *n* represent independent biological replicates. If needed, data were logarithmically transformed to correct for unequal variance or non-normal distribution. No outliers were excluded from the statistical analyses. All statistical analyses and figures were generated with GraphPad Prism 4 (La Jolla, CA, United States).

For statistical analysis of metabolomics data, annotated metabolites were used for multivariate statistical analysis. Pathway analyses and Principal Component Analysis (PCA) plots were generated with MetaboAnalyst 3.0 (Xia and Wishart, 2016). The significance of individual metabolites between the four treatment groups was assessed with a one-way ANOVA followed by Fisher’s *post hoc* analysis and Holm FDR-correction, with a *p*-value of <0.05 indicating significance (*n* = 6/group). If needed, data were logarithmically transformed to correct for unequal variance or non-normal distribution. No outliers were excluded from the statistical analyses. Figures were generated with GraphPad Prism 4 (La Jolla, CA, United States), PowerPoint 2016 (Microsoft, Redmond, WA, United States), and MetaboAnalyst 3.0.

## Results

### Nitrate Tolerance in LLC-PK1 Cells

**FIGURE 2:**
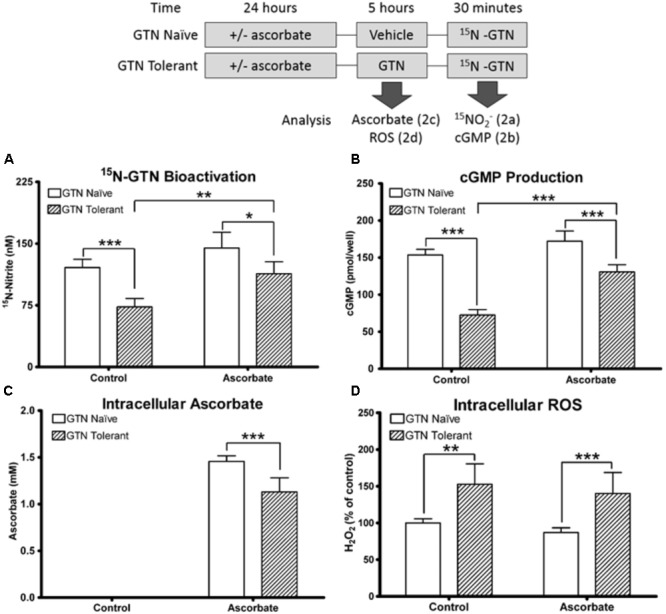
Nitrate tolerance in LLC-PK1 cells. Cells were pre-treated with vehicle control (GTN Naïve) or GTN (GTN Tolerant). **(A)** Pre-treatment with GTN caused reduced metabolism of ^15^N_3_-GTN into ^15^N-nitrite (*p* < 0.001), which was attenuated by ascorbic acid (*p* < 0.01). **(B)** cGMP production was reduced by GTN (*p* < 0.001), and attenuated with ascorbic acid (*p* < 0.001). **(C)** Non-supplemented cells had no detectable ascorbic acid. Ascorbic acid-treated cells had 1.46 ± 0.03 mM intracellular ascorbic acid. GTN decreased ascorbic acid (*p* < 0.001). **(D)** GTN increased H_2_O_2_ in both control (*p* < 0.01) and ascorbic acid-treated cells (*p* < 0.001). One-way ANOVA with Bonferroni *post hoc* analysis, *p* < 0.05 indicating significance (*n* = 6/group), GraphPad Prism 4. ^∗^*P* < 0.05, ^∗∗^*P* < 0.01, ^∗∗∗^*P* < 0.001.

We induced nitrate tolerance in ascorbic acid-deficient or ascorbic acid-supplemented LLC-PK1 cells with a 5-h pre-treatment of 1 μM GTN. Pre-treatment with GTN (GTN Tolerant) caused reduced bioactivation of a second challenge with ^15^N_3_-GTN, as evidenced by quantification of ^15^N-nitrite (*p* < 0.001). This observed nitrate tolerance was attenuated by co-treatment with ascorbic acid (*p* < 0.01). There was no significant difference in ^15^N_3_-GTN bioactivation between control cells and cells pre-treated with both GTN and ascorbic acid. In cells that did not receive a pre-treatment with GTN (GTN Naïve), ascorbic acid did not cause a significant increase in ^15^N_3_-GTN bioactivation (**Figure 2A**).

Cyclic guanosine-3′,-5′-monophosphate (cGMP) is released when NO interacts with soluble guanylate cyclase (sGC), and is a key intermediate in NO-induced vasodilation. cGMP was measured as a biomarker of NO production. Pre-treatment with GTN caused impaired bioactivation of GTN into NO (*p* < 0.001), which was attenuated by co-treatment with ascorbic acid (*p* < 0.001; **Figure 2B**). There was no significant difference in cGMP production between control cells and cells treated with both GTN and ascorbic acid. The cGMP results are supported by studies demonstrating that cGMP can be measured as a marker of nitrate tolerance, and that tolerance can be induced in LLC-PK1 cells with GTN treatment (Watanabe et al., 1993; Hinz and Schröder, 1998b). These results demonstrate that ascorbic acid increases both nitrite and NO production from GTN in nitrate tolerant cells.

We quantified intracellular ascorbic acid to determine the interactions between ascorbic acid and GTN. Intracellular concentration of ascorbic acid in the supplemented cells was 1.46 ± 0.03 mM. Ascorbic acid was not detected in the non-supplemented cells (lower limit of detection 1 μM). The non-detectable basal levels in these cultured porcine cells reflect that ascorbate-producing mammals synthesize ascorbic acid primarily in the liver (Drouin et al., 2011). The intracellular concentration exceeded the 100 μM treatment concentration due to active uptake of ascorbic acid. Nitrate tolerant cells had reduced intracellular ascorbic acid compared to cells treated with ascorbic acid alone (1.07 ± 0.06 mM; *p* < 0.001; **Figure 2C**). A 2′-7′-dichlorofluorescein diacetate (DCFDA) assay to measure intracellular H_2_O_2_ demonstrated that GTN caused increased intracellular oxidative stress (*p* < 0.01), which was not attenuated by ascorbic acid supplementation (**Figure 2D**). The increase in oxidative stress can be caused by superoxide ($O_{2}^{–}$) production by XO, mitochondrial dysfunction, or formation of peroxynitrite (ONOO^-^) from the reaction between NO and $O_{2}^{–}$. These results support the “oxidative stress hypothesis” by Thomas Münzel, which postulates that oxidative stress caused by nitrate treatment leads to enzyme inactivation (Münzel et al., 1995). These results suggest that the protective role of ascorbic acid may not be solely due to its antioxidant capacity.

### Untargeted Metabolomics of LLC-PK1 Cells Exposed to GTN

Metabolomics analysis was performed on nitrate tolerant and control LLC-PK1 cells that were ascorbic acid-deficient or ascorbic acid-supplemented. Eighty-five metabolites were assigned using our in-house library, of which 35 were significantly different in at least one of the groups compared to the others (One-way ANOVA with Fisher’s *Post hoc* analysis; *n* = 6/group; *p* < 0.05 after Holm’s FDR-correction for multiple testing, MetaboAnalyst 3.0). All annotated metabolites are listed in the **Supplementary Material** with their molecular formula, accurate mass, ppm error, and retention time (**Supplementary Table 1**). The *p*-values for all annotated metabolites between all treatment groups are listed in the **Supplementary Information** (**Supplementary Table 2**).

### Multivariate Analysis

**FIGURE 3:**
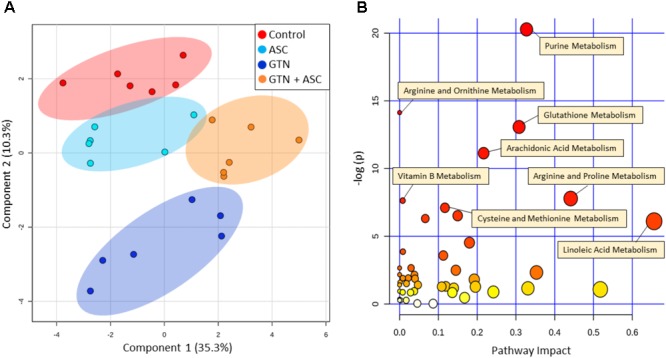
Multivariate analysis of metabolomics data. Metabolomics analysis was performed on control, ascorbic acid (ASC), glyceryl trinitrate (GTN), and GTN + ASC groups. **(A)** Principal Component Analysis (PCA) score plot, and **(B)** Pathway Analysis. Analyses are based on all annotated metabolites (*n* = 6/group; log transformation, Pareto scaling). Pathway analysis tabulated results are in **Supplementary Table 3**. Plots were generated with MetaboAnalyst 3.0.

Principal Component Analysis plots of all annotated metabolites show separation and clustering of control, GTN, ASC, and GTN + ASC groups (**Figure 3A**). The PCA plots demonstrate that the treatments had a significant effect on the metabolic profile, and that the replicates cluster together. MarkerView software was used to generate log fold-change vs. *p*-value “Volcano” plots for all features detected in positive ion mode (**Supplementary Figure 2**). Plotting control and GTN-treated cells resulted in a total of 653 significantly changed features (*p* < 0.05). Ascorbic acid treatment resulted in a modest 41 significantly changed features. However, of the 601 features significantly increased with GTN treatment (*p* < 0.05, log fold-change > 0.2), 248 were decreased with ascorbic acid co-treatment (*p* < 0.05, log fold-change > 0.2). These results indicate that ascorbic acid treatment alone had a moderate effect on the metabolic profile, yet it prevented many of the GTN-induced metabolic changes.

### Pathway Analysis

**FIGURE 4:**
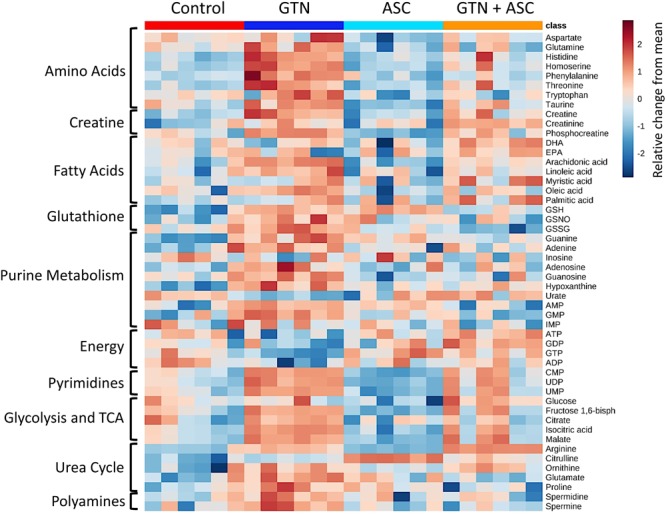
Heat map of metabolite abundance per treatment group. Metabolomics analysis was performed on control, ascorbic acid (ASC), glyceryl trinitrate (GTN), and GTN + ASC groups (*n* = 6/group). The metabolites were chosen based on top p-values and physiological significance. Colors indicate *z*-score (standard deviation from the mean). The heat map was generated with MetaboAnalyst 3.0 using normalized data (log transformation, Pareto scaling) using Euclidean distance measure.

MetaboAnalyst was used to generate a pathway analysis with the annotated metabolites (**Figure 3B**). Eleven pathways were significantly changed upon GTN treatment compared to control (*p* < 0.05 after Holm FDR correction; tabulated results and *p*-values available in **Supplementary Table 3**). Of the significant pathways, most notable were purine metabolism, arginine and ornithine metabolism, glutathione metabolism, cysteine and methionine metabolism, arginine and proline metabolism, arachidonic metabolism, and linoleic acid metabolism. Furthermore, MetaboAnalyst was used to generate a heat map of select metabolites within pathways that are changed among Control, GTN, ASC, and GTN + ASC groups (**Figure 4**).

**FIGURE 5:**
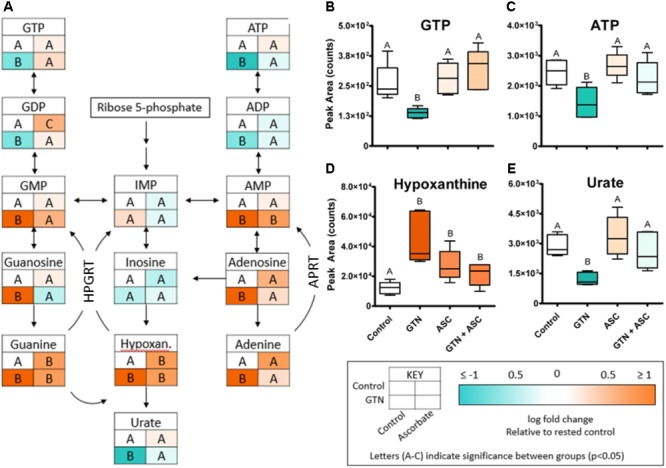
GTN disrupts purine metabolism. **(A)** GTN treatment resulted in increases of the levels the purines GMP, guanosine, guanine, AMP, adenosine, and adenine; GTN treatment resulted in decreases of nucleotide diphosphates and triphosphates. **(B)** GTP and **(C)** ATP are decreased with GTN treatment, which was prevented by ascorbic acid. **(D)** Hypoxanthine was significantly increased with GTN treatment. **(E)** Uric acid was significantly decreased with GTN treatment, which was prevented with ascorbic acid co-treatment. One-way ANOVA with Fisher’s *post hoc* analysis and FDR correction, *p* < 0.05 indicating significance (*n* = 6/group), GraphPad Prism 4. *P*-values for all annotated metabolites are located in **Supplementary Table 2**. HPGRT, hypoxanthine/guanine phosphoribosyl transferase; APRT, adenine phosphoribosyl transferase.

Glyceryl trinitrate treatment impaired purine metabolism and energy production (**Figure 5**). Fatty acids (oleic acid, linoleic acid, arachidonic acid, myristic acid, palmitic acid), glycolytic intermediates (glucose, fructose 1,6-bisphosphate) and TCA cycle intermediates (citrate, isocitrate, malate) were increased with GTN treatment. Several purines were significantly increased in GTN-treated cells (**Figure 5A**). This pattern is observed in guanine, guanosine, and guanosine monophosphate (GMP) and well as in adenine, adenosine, and adenosine monophosphate (AMP). The only purines that were not significantly increased with GTN treatment were inosine and inosine monophosphate (IMP). Interestingly, we observed that increased AMP and GMP levels do not correlate to increased ADP/ATP or GDP/GTP. An increase in the AMP/ADP/ATP ratio, otherwise known as the adenylate energy charge, indicates significant mitochondrial dysfunction potentially due to the increase in oxidative stress resulting from GTN treatment.

Our data further suggest that ascorbic acid prevents the GTN-induced inactivation of XO. XO catalyzes the oxidative conversion of hypoxanthine and xanthine into uric acid, which are the final steps in purine metabolism in humans. In GTN-treated cells, hypoxanthine was increased (*p* < 0.001) and uric acid was decreased (*p* < 0.05; **Figures 5D,E**). These results suggest that XO activity is impaired with GTN treatment. Co-treatment with ascorbic acid prevented this effect, resulting in increased metabolism of hypoxanthine to uric acid and restoring purine metabolism.

**FIGURE 6:**
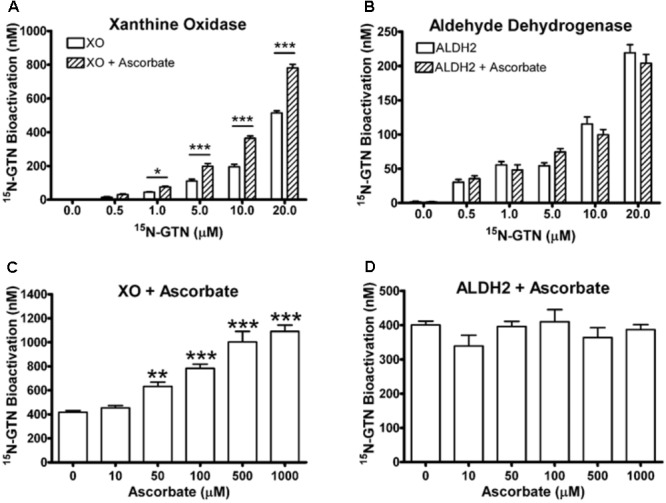
Xanthine oxidase (XO) and mitochondrial aldehyde dehydrogenase (ALDH2) assays. **(A,B)** XO and ALDH2 bioactivated ^15^N_3_-GTN (0.5–20 μM) into ^15^N-nitrite with a linear dose-dependent response (*R*^2^ > 0.99, *p* < 0.001 with Linear Fit). 100 μM sodium ascorbate increased activity of XO, but not ALDH2. **(C,D)** XO and ALDH2 were incubated with ^15^N_3_-GTN (20 μM) and sodium ascorbate (10 – 1000 μM). Ascorbic acid increased GTN bioactivation by XO at all concentrations above 10 μM (*p* < 0.05), yet did not change GTN bioactivation by ALDH2 at any concentration. One-way ANOVA with Bonferroni *post hoc* analysis, *p* < 0.05 indicating significance (*n* = 3/group), GraphPad Prism 4. ^∗^*P* < 0.05, ^∗∗^*P* < 0.01, ^∗∗∗^*P* < 0.001.

To validate our metabolomics results, we incubated commercially available bovine XO and human recombinant ALDH2 with ^15^N_3_-GTN (0.5 – 20 μM). The enzymes were incubated in HEPES buffer (pH 7.4) for 30 min at 37°C. ALDH2 (10 μg/mL, containing 100 μM DTT) was incubated with 100 μM NAD+ and XO (0.8 U/mL) was incubated with 100 μM xanthine. Samples were assayed for ^15^N-nitrite to measure ^15^N_3_-GTN bioactivation. We determined that both XO and ALDH2 can bioactivate GTN into nitrite with a linear dose-response (**Figures 6A,B**). Ascorbic acid (100 μM) increased GTN bioactivation by XO, but not ALDH2. XO and ALDH2 were incubated with ^15^N_3_-GTN (20 μM) and sodium ascorbate (10 – 1000 μM) to characterize the dose-response of ascorbic acid. Ascorbic acid increased GTN bioactivation by XO at concentrations 50 μM and greater (*p* < 0.01), but did not have an effect on ALDH2 at any concentration (**Figures 6C,D**). The effect of ascorbic acid on XO-mediated GTN bioactivation is physiologically relevant given that intracellular ascorbic acid was 1.46 ± 0.03 mM in LLC-PK1 cells.

**FIGURE 7:**
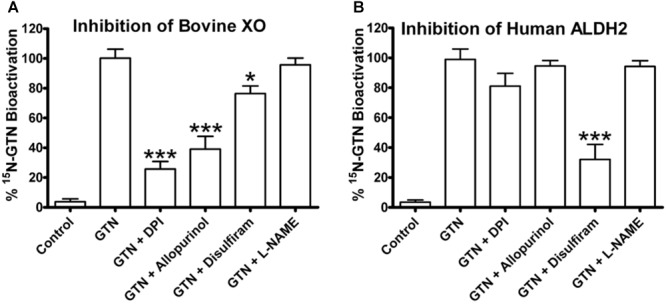
Inhibitors of bovine XO and human ALDH2. XO and ALDH2 were incubated with ^15^N_3_-GTN (20 μM) and allopurinol, diphenylene iodonium (DPI), disulfiram, and L-N^G^-Nitroarginine methyl ester (L-NAME) (100 μM). **(A)** XO was inhibited by DPI (*p* < 0.001), allopurinol (*p* < 0.001), and disulfiram (*p* < 0.05). **(B)** ALDH2 was inhibited by disulfiram (*p* < 0.001). One-way ANOVA with Bonferroni *post hoc* analysis, *p* < 0.05 indicating significance (*n* = 3/group), GraphPad Prism 4. ^∗^*P* < 0.05, ^∗∗^*P* < 0.01, ^∗∗∗^*P* < 0.001.

Inhibition of bovine XO with the inhibitors allopurinol and diphenyleneiodonium chloride (DPI) significantly decreased GTN bioactivation (**Figure 7**; *p* < 0.001). Allopurinol is a specific molybdenum-site inhibitor, whereas DPI is a non-specific FAD-site inhibitor of XO (Li et al., 2001). ALDH2 was inhibited by disulfiram (*p* < 0.001). Interestingly, disulfiram decreased GTN bioactivation by XO (*p* < 0.05), demonstrating off-target effects. The eNOS inhibitor L-NAME did not have a significant effect on ALDH2 or XO.

**FIGURE 8:**
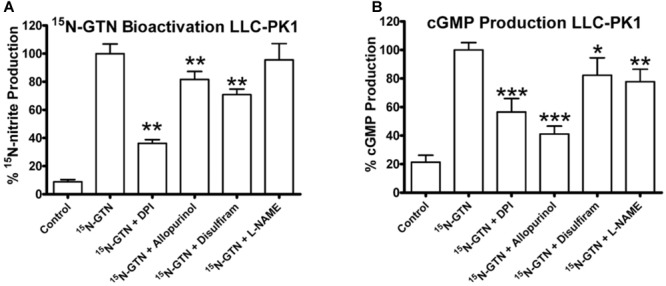
Inhibition of XO and ALDH2 in LLC-PK1 cells. LLC-PK1 cells were incubated with ^15^N-GTN (1 μM) in the presence/absence of allopurinol, diphenylene iodonium (DPI), disulfiram, and L-N^G^-Nitroarginine methyl ester (L-NAME) (100 μM). **(A)** GTN bioactivation was inhibited by DPI (*p* < 0.01), allopurinol (*p* < 0.01), and disulfiram (*p* < 0.01). **(B)** cGMP production was inhibited by DPI (*p* < 0.001), allopurinol (*p* < 0.001), disulfiram (*p* < 0.05), and L-NAME (*p* < 0.01). One-way ANOVA with Bonferroni *post hoc* analysis, *p* < 0.05 indicating significance (*n* = 6/group), GraphPad Prism 4. ^∗^*P* < 0.05, ^∗∗^*P* < 0.01, ^∗∗∗^*P* < 0.001.

We then used the chemical inhibitors of XO and ALDH2 to test the role of XO and ALDH2 on GTN bioactivation in LLC-PK1 cells. It should be noted that with a single dose of the inhibitors, we were not attempting to characterize enzyme kinetics, but testing for presence or absence of XO and ALDH2 activity in the LLC-PK1 cells. Both ^15^N-nitrite production and cGMP production were reduced upon treatment with XO inhibitors, allopurinol and DPI (**Figure 8**). Disulfiram caused a minor but significant decrease in both GTN bioactivation and cGMP production (*p* < 0.01, *p* < 0.05). Together, these results demonstrate that both XO and ALDH2 bioactivate GTN in LLC-PK1 cells.

We measured XO and ALDH2 enzyme activity in LLC-PK1 cells. LLC-PK1 cells were supplemented with 100 μM ascorbic acid for 24 h, then subjected to a 5-h 1 μM GTN treatment following the same protocol performed for the metabolomics analysis. XO activity was measured with a commercially available assay (Xanthine Oxidase Activity Assay Kit, ABcam, Cambridge, United Kingdom), which measured XO-mediated oxidation of xanthine, thereby converting O_2_ to H_2_O_2_ which reacts stoichiometrically with an OxiRed Probe (Ex/Em = 535/587 nm). ALDH2 was measured with a commercially available assay (ALDH2 Activity Assay Kit, Abcam, Cambridge, United Kingdom) which uses immunocapture to isolate ALDH2 in a microplate and measures the acetaldehyde-dependent conversion of NAD+ into NADH, which reacts with a reporter dye (absorbance 450 nm).

**FIGURE 9:**
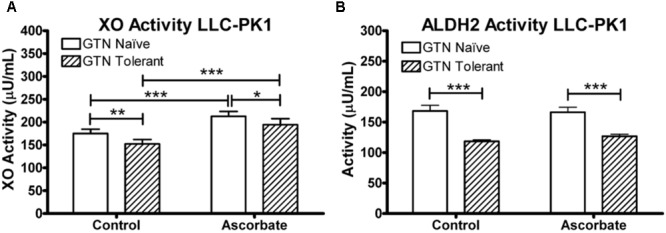
XO and ALDH2 activity in LLC-PK1 cells. Cells were incubated in the presence or absence of sodium ascorbate (100 μM), then treated with 1 μM GTN. Cells were analyzed for XO (xanthine conversion producing H_2_O_2_) or ALDH2 (acetaldehyde conversion producing NADH) activity. **(A)** GTN decreased XO activity (*p* < 0.05), and ascorbic acid increased XO activity (*p* < 0.001). The GTN with ascorbic acid group had significantly higher XO activity compared to control, demonstrating that ascorbic acid prevented GTN-induced XO inactivation (*p* < 0.001). **(B)** GTN decreased ALDH2 activity (*p* < 0.001). Ascorbic acid did not change ALDH2 activity. One-way ANOVA with Bonferroni *post hoc* analysis, *p* < 0.05 indicating significance (*n* = 6/group), GraphPad Prism 4. ^∗^*P* < 0.05, ^∗∗^*P* < 0.01, ^∗∗∗^*P* < 0.001.

LLC-PK1 cells express active XO and ALDH2. XO was inactivated by GTN (*p* < 0.01), which was attenuated by ascorbic acid co-treatment (**Figure 9**; *p* < 0.001). Ascorbic acid increased XO activity in both GTN naïve and GTN tolerant cells (*p* < 0.001). ALDH2 activity was reduced by GTN treatment (*p* < 0.001), however, ascorbic acid did not alter ALDH2 activity.

## Discussion

We selected the porcine renal epithelial LLC-PK1 cell line for our studies because this cell line expresses XO and has robust NO-sGC-cGMP biosynthetic capacity, unlike EA.hy926 human endothelial cells, which we used in our previous work (Axton et al., 2016). The LLC-PK1 cell line also expresses NO synthase (Tracey et al., 1994; Healy et al., 2000; Lima et al., 2001). We pre-treated LLC-PK1 cells with GTN for 5 h as a cell culture model of nitrate tolerance. The data presented in **Figure 2** show that GTN pre-treatment resulted in reduced ^15^${\mathrm{NO}}_{2}^{–}$ production from GTN upon a second challenge with ^15^N-labeled GTN, which we quantified as an index of nitrate tolerance. When cells were cultured in the presence of ascorbic acid and subsequently pre-treated with GTN, no reduction of ^15^${\mathrm{NO}}_{2}^{–}$ or cGMP formation was observed upon ^15^N-GTN challenge compared to GTN-naïve, ascorbate-deficient cells. This finding demonstrates that ascorbic acid supplementation can attenuate GTN-induced nitrate tolerance. Our finding that GTN-pretreatment resulted in lower intracellular concentrations of ascorbic acid (**Figure 2C**) is in agreement with previously reported lower plasma ascorbic levels in guinea pigs treated with GTN (Wenzl et al., 2009). This is a relevant finding because humans and guinea pigs, unlike most other animals, are unable to synthesize ascorbic acid and require it in the diet.

The untargeted metabolomics experiments revealed that GTN affects several important metabolic pathways involved in maintaining redox homeostasis, amino acid metabolism, and purine metabolism (**Figure 3**). Glutathione (GSH) levels were elevated following both GTN treatment (*p* < 0.01) and ascorbic acid supplementation (*p* < 0.05; **Figure 4** and **Supplementary Table 2**). This may be due to an adaptive stress response to the reactive oxygen and nitrogen species generated upon GTN treatment. Alternatively, peroxynitrite produced by GTN-bioactivation causes *S*-nitrosylation of glutathione *S*-transferase (GST) (Ji and Bennett, 2003), potentially causing reduced conjugation of GSH for detoxification. Ascorbic acid may increase intracellular glutathione through a sparing mechanism. GSSG is generated when GSH reacts with ROS. The ratio of GSH/GSSG is considered a biomarker of oxidative stress. Cells treated with GTN have a significant increase in GSSG (*p* < 0.001), which is prevented by ascorbic acid supplementation (*p* < 0.001; **Figure 4**). These findings demonstrate that ascorbic acid supplementation normalizes glutathione metabolism in this cell culture model of nitrate tolerance.

In addition, we found that GTN impairs L-arginine metabolism (**Figure 4**). eNOS metabolizes L-arginine and O_2_ into L-citrulline and NO. GTN treatment increases L-arginine (*p* < 0.001) and decreases L-citrulline (*p* < 0.01). This accumulation of L-arginine suggests that eNOS activity is impaired with GTN treatment. *In vitro* studies have shown that eNOS has higher expression but lower activity after GTN treatment, and is associated with higher superoxide levels (Münzel et al., 1995). eNOS activity may be lower because peroxynitrite, a product of enzymatic GTN bioactivation, can oxidize tetrahydrobiopterin (BH4), an essential cofactor of eNOS activity, into the inactive dihydrobiopterin (BH2) via the radical intermediate trihydrobiopterin (BH3) (Laursen et al., 2001). Ascorbic acid can recycle BH3 back into BH4, effectively restoring eNOS activity (Kuzkaya et al., 2003). Citrulline is significantly increased with ascorbic acid treatment (*p* < 0.001), suggesting that ascorbic acid is increasing eNOS activity.

Accumulation of L-arginine has significant downstream metabolic effects on the urea cycle and polyamine metabolism. Ornithine levels were higher in GTN-treated cells compared to the controls (*p* < 0.001; **Figure 4** and **Supplementary Table 2**). Increased ornithine, a member of the urea cycle, results in changes in amino acid metabolism, TCA cycle, glycolysis, and gluconeogenesis. We observed that increased ornithine parallels increased proline and glutamate, which are products of ornithine. Glutamate is an amino acid that can feed into the TCA cycle via reversible transamination or glutamate dehydrogenase. The polyamines spermine and spermidine are also increased with GTN treatment (*p* < 0.05, *p* < 0.05). Polyamines control cell growth and differentiation and are important modulators of ion channels such as NMDA (*N*-methyl D-aspartate receptor) and AMPA (α-amino-3-hydroxy-5-methyl-4-isoxazolepropionic acid receptor).

The observed depletion of ADP and ATP points to GTN-induced disruption of purine metabolism (**Figure 5**) and mitochondrial dysfunction. ALDH2, a mitochondrial GTN-bioactivating enzyme, can release peroxynitrite when exposed to GTN (Oelze et al., 2011). High levels of nitrate lead to mitochondrial uncoupling, perhaps mediated by ALDH2 activity (Needleman and Hunter, 1966). In addition, NO modulates cytochrome c oxidase (Complex IV of mitochondrial respiration) via *S*-nitrosylation (Shiva et al., 2005). Together, these findings indicate that GTN, or products thereof, are causing mitochondrial dysfunction and reduced ATP production. Interestingly, we observed that ascorbic acid co-treatment restored ATP and ADP production, as well as GDP and GTP (**Figures 5B,C**). Although kinetic considerations argue against ascorbic acid being able to prevent the formation of peroxynitrite, it is conceivable that ascorbic acid scavenges secondary oxidizing species derived from peroxynitrite (Carballal et al., 2014). This would reduce mitochondrial oxidative stress and maintain cellular ATP production. Ascorbic acid has previously been shown to detoxify and eliminate 4-hydroxy-2(*E*)-nonenal to prevent mitochondrial dysfunction in human monocytic THP-1 cells (Miranda et al., 2009).

Our data further indicate that ascorbic acid prevents the GTN-induced inactivation of XO (**Figure 5**). Since both hypoxanthine and xanthine undergo oxidation at the molybdenum site of XO, this suggests that ascorbic acid may act through the molybdenum site to increase XO activity. Inactivation of XO could contribute to the increased levels of purines, including AMP and GMP. The nucleotide salvage pathways hypoxanthine/guanine phosphoribosyl transferase (HPGRT) and adenine phosphoribosyl transferase (APRT) can recover the bases hypoxanthine, adenine, and guanine back into their corresponding nucleotide monophosphates (**Figure 5**). Therefore, XO inactivation and disruption of the final step of purine metabolism could lead to accumulation of purines and nucleotide monophosphates.

An alternate explanation for the increase in purines is that decreased ATP would lead to increase of adenylate kinase (ADK, myokinase) activity, disproportioning 2 ADP to produce ATP + AMP. Increased activity of ADK would lead to depletion of ADP and an increase in ATP and AMP. With increased nucleotide monophosphates we would also expect to see an increase in the corresponding bases (hypoxanthine, adenine, and guanine). Interestingly, ascorbic acid deficiency has been previously shown to activate the purine nucleotide cycle in zebrafish by increasing AMP deaminase (AMPD) activity (Kirkwood et al., 2012). The absence of a significant change in IMP and inosine with treatment suggests low AMPD activity, with most of the AMP either being metabolized into adenosine and adenine, or being utilized for ATP production. Ascorbic acid deficiency has previously been shown to decrease the adenylate charge in zebrafish (Kirkwood et al., 2012) and in chick cartilage cells (Shapiro et al., 1991). We demonstrate here that GTN-induced decrease of the adenylate energy charge is prevented by ascorbic acid co-treatment.

Our metabolomics results suggest both mitochondrial dysfunction and disrupted purine metabolism. Both of these dramatic metabolic changes were prevented with ascorbic acid co-treatment. These metabolic effects can be attributed to impaired XO and ALDH2 activity, both GTN-bioactivating enzymes that are known to be inactivated with continuous GTN treatment. The precise mechanism of GTN bioactivation has yet to be elucidated. It is commonly believed that ALDH2 bioactivates GTN into nitrite in endothelial cells, which is then reduced to NO (Chen et al., 2002). NO diffuses into the smooth muscle cells, which generally do not produce NO themselves, where a cascade of signaling events activates sGC to produce cyclic guanosine-3′,-5′-monophosphate (cGMP) to induce vasodilation (Rychter et al., 2016). Another enzyme that has been shown to bioactivate both inorganic and organic nitrates into NO is XO (Millar et al., 1998; Doel et al., 2000; Godber et al., 2000, 2001), however, the physiological significance has yet to be demonstrated. Although XO is inactivated by continuous GTN treatment, it is not yet known if XO is an important GTN bioactivating enzyme *in vivo*. It is believed that XO is a low-affinity pathway of GTN bioactivation that may be more significant in certain disease states and under low oxygen ischemic conditions (Münzel et al., 2005).

ALDH2 is widely regarded as the primary GTN-bioactivating enzyme *in vivo*. However, our metabolomics results suggest a role of XO in the development of nitrate tolerance. Of particular importance, the protective role of ascorbic acid may be mediated through XO. We chose to use chemical inhibitors of both XO and ALDH2 to determine relative role of these enzymes in GTN bioactivation in LLC-PK1 cells. To determine the specificity of the inhibitors, we first incubated bovine XO and human ALDH2 with the inhibitors allopurinol (inhibitor of molybdenum site of XO), disulfiram (ALDH2 inhibitor), diphenyleneiodonium (FAD-site inhibitor of flavoenzymes), and L-N^G^-Nitroarginine methyl ester (L-NAME, inhibitor of eNOS). This approach was chosen because knockouts and knockdowns of XOR in LLC-PK1 cells were not viable, which was not unexpected given that mouse knockouts of XOR are lethal (Ohtsubo et al., 2004).

The observed reduction in XO and ALDH2 activity by GTN (**Figure 9**) appears have an impact on several metabolic pathways, most notably purine metabolism (**Figure 5**). Inactivation of XO by oxidative species is irreversible and activity can only be recovered by *de novo* protein synthesis (Hinz and Schröder, 1998a). Our studies suggest that ascorbic acid has the ability to prevent nitrate tolerance by protecting XO, but not ALDH2, from GTN-induced inactivation (**Figure 9**). Importantly, ascorbic acid supplementation may counteract GTN-induced adverse effects on metabolic pathways that are unrelated to NO-mediated vasodilation.

Proposing that inactivation of XO is the cause of nitrate tolerance is not without controversy. Most textbooks and publications claim that ALDH2 is the key GTN-bioactivating enzyme, but our results, and findings published by others, suggest otherwise. The role of ALDH2 in GTN bioactivation has been recently questioned because siRNA-mediated knockouts and overexpression of ALDH2 had no significant effect on cGMP production in porcine epithelial cells (D’souza et al., 2013). It has been proposed that ALDH2 acts as a scavenger of HNE and other reactive species, but in fact does not directly metabolize GTN (D’souza et al., 2014). If ALDH2 activity is impaired, oxidative stress levels will rise and lead to inactivation of XO and reduced GTN bioactivation. ALDH2 and XO activity are therefore linked, so studies on the development of nitrate tolerance should account for bioactivation from both enzymes. Our results demonstrate that both ALDH2 and XO are involved in GTN bioactivation. ALDH2 catalyzes the high-affinity pathway of GTN bioactivation into nitrite (Beretta et al., 2012), however, when it is inactivated and intracellular oxidative stress levels rise, XO could become the predominant GTN-bioactivating enzyme as a second line of defense. Given that XO is necessary to convert nitrite into NO, preserving XO activity is paramount to preventing nitrate tolerance. Our mechanistic studies provide a plausible explanation for published observations that co-treatments of GTN with ascorbic acid prevent the development of nitrate tolerance (Bassenge and Fink, 1996; Bassenge et al., 1998; Watanabe et al., 1998).

## Data Availability Statement

All datasets generated for this study are included in the manuscript and in the Supplementary Files.

## Author Contributions

EA and JS designed the experiments. EA, EC, and CM performed the cell culture and enzyme experiments. EA and JC performed the mass spectrometry analyses. JS provided technical and material support. EA and JS wrote the manuscript, and all authors reviewed the manuscript.

## Conflict of Interest Statement

The authors declare that the research was conducted in the absence of any commercial or financial relationships that could be construed as a potential conflict of interest.
