# Supplementary material for: Metabolomics-Driven Elucidation of Cellular Nitrate Tolerance Reveals Ascorbic Acid Prevents Nitroglycerin-Induced Inactivation of Xanthine Oxidase
Source: Front Pharmacol. 2018 Sep 25;9:1085. doi: 10.3389/fphar.2018.01085 (PMC6167911; doi:10.3389/fphar.2018.01085)
Supplement: Supplementary file 1 [file Table_1.DOCX]

**Ascorbic Acid Prevents Nitroglycerin-Induced Inactivation of Xanthine Oxidase in a Cell Culture Model of Nitrate Tolerance**

Elizabeth R. Axton^1,2,3,4^, Jan F. Stevens^1,2*^

^1^The Linus Pauling Institute, Oregon State University, Corvallis, Oregon, U.S.A.

^2^Department of Pharmaceutical Sciences, Oregon State University, Corvallis, Oregon, U.S.A

^3^Department of Environmental and Molecular Toxicology^3^, Oregon State University, Corvallis, Oregon, U.S.A.

^4^Current address: Jackson Laboratories, Sacramento, California, U.S.A.

**Supplementary Information** consisting of Supplementary Figure 1, Supplementary Figure 2, Supplementary Table1, Supplementary Table 2, and Supplementary Table 3.


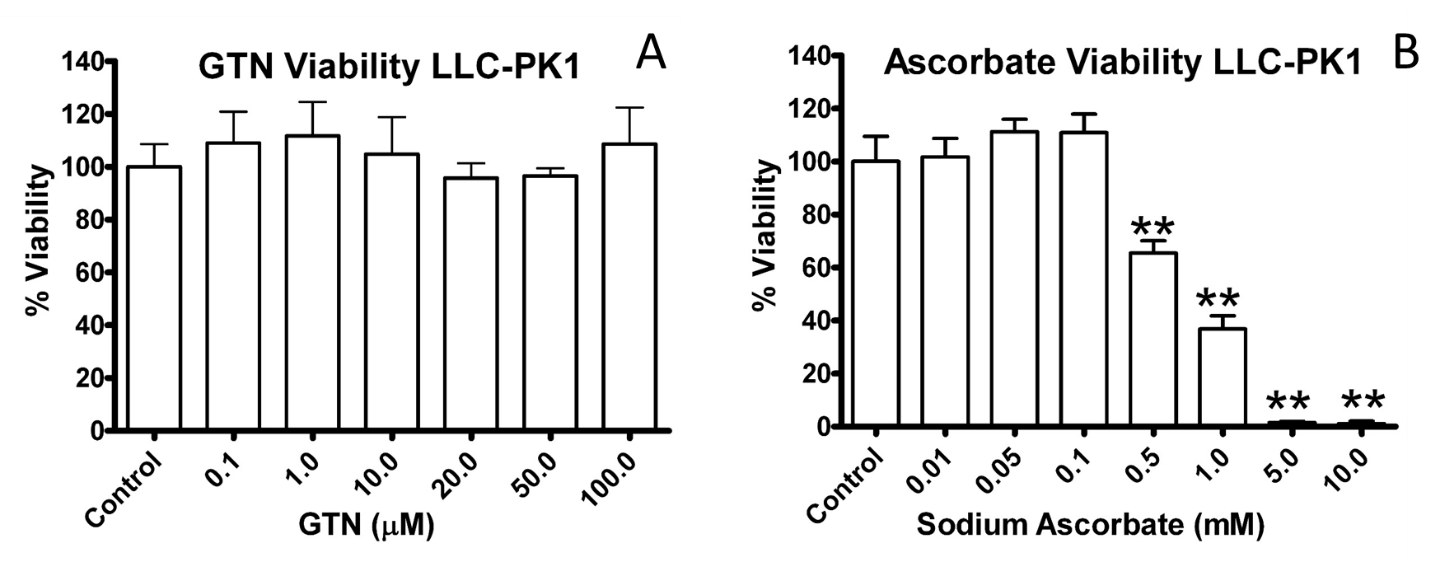


SUPPLEMENTARY FIGURE 1. MTT Assay of LLC-PK1 cells. A) GTN did not cause any change in viability at any concentration. B) sodium ascorbate reduced cell viability 0.5 mM and higher (P<0.01). One-way ANOVA with Bonferroni post-hoc analysis, P<0.05 indicating significance (n=5/group), GraphPad Prism 4.


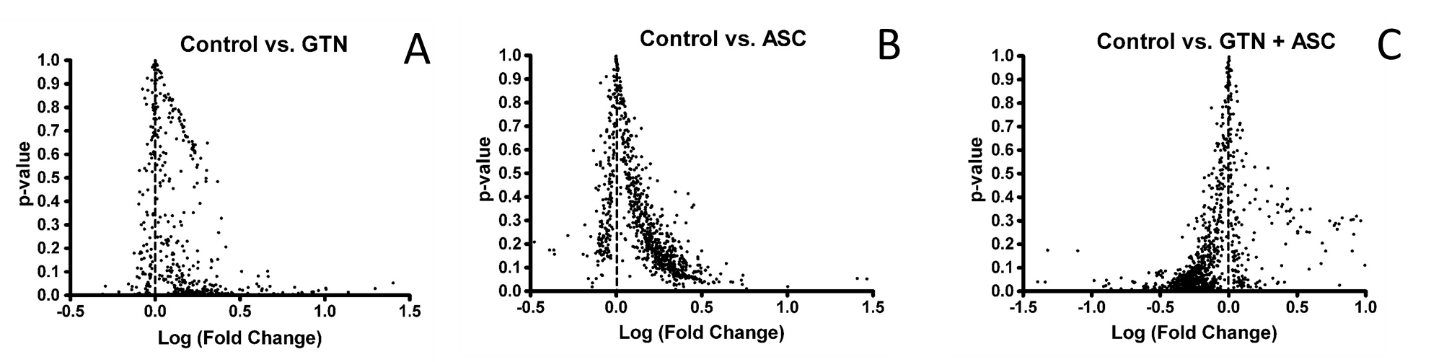


SUPPLEMENTARY FIGURE 2. Log (fold change) vs. p-value “volcano” plots. Group comparisons are based on differences in the abundance of spectral features detected in the positive ion mode (shown as black dots). Log 10 transformed fold-changes were calculated from an average of 6 samples per treatment group by MarkerView software. Figures were generated with GraphPad Prism 4.


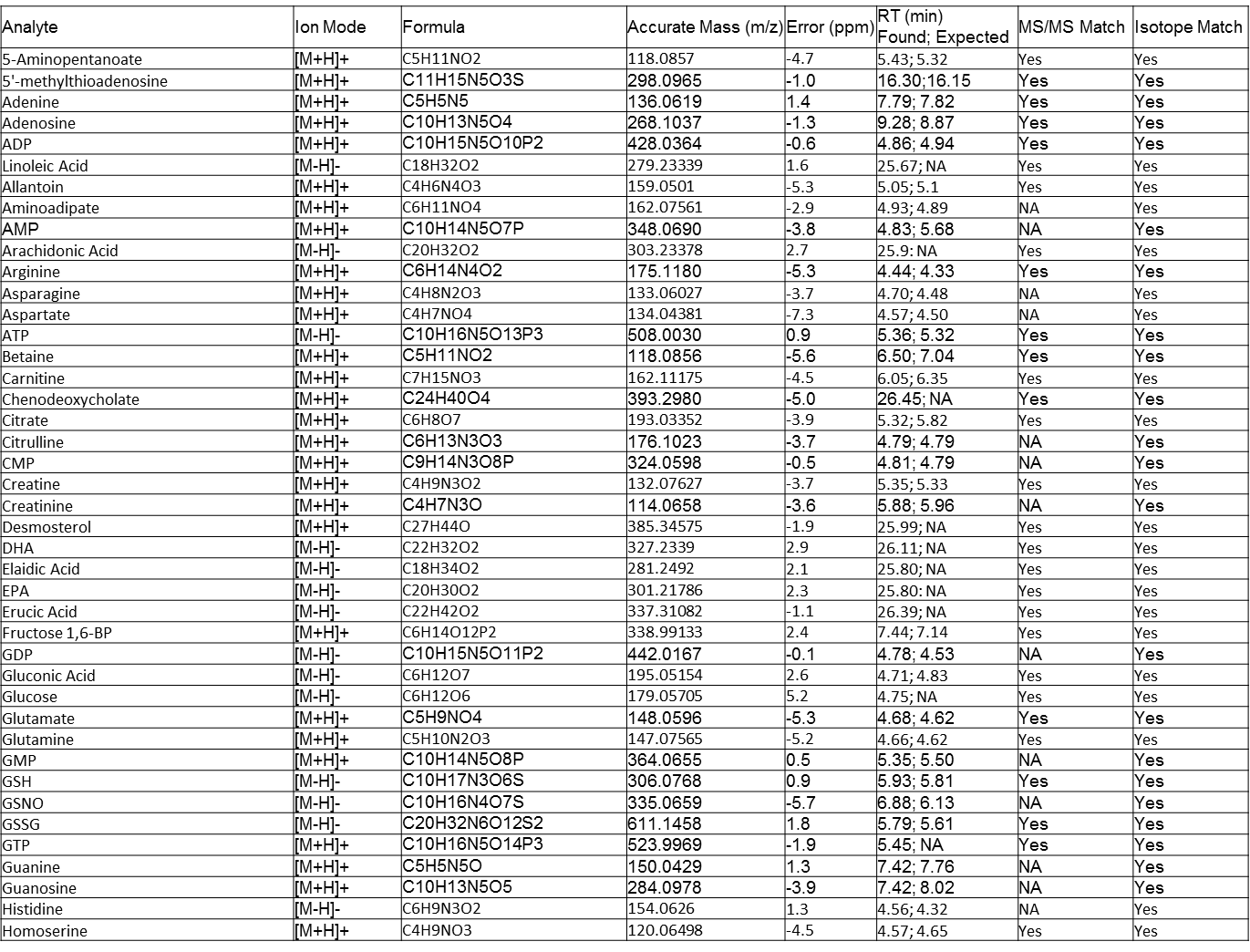
SUPPLEMENTARY TABLE 1. Metabolites detected in LLC-PK1 cells. 85 metabolites were annotated. Identifiers (accurate mass, ppm error, retention time (RT), and isotope and MS/MS matching) were acquired using MasterView with the retention-time matched IROA library. NA – not acquired, or unknown. Metabolites were excluded if they did not have either MS/MS fragmentation or retention time matching.


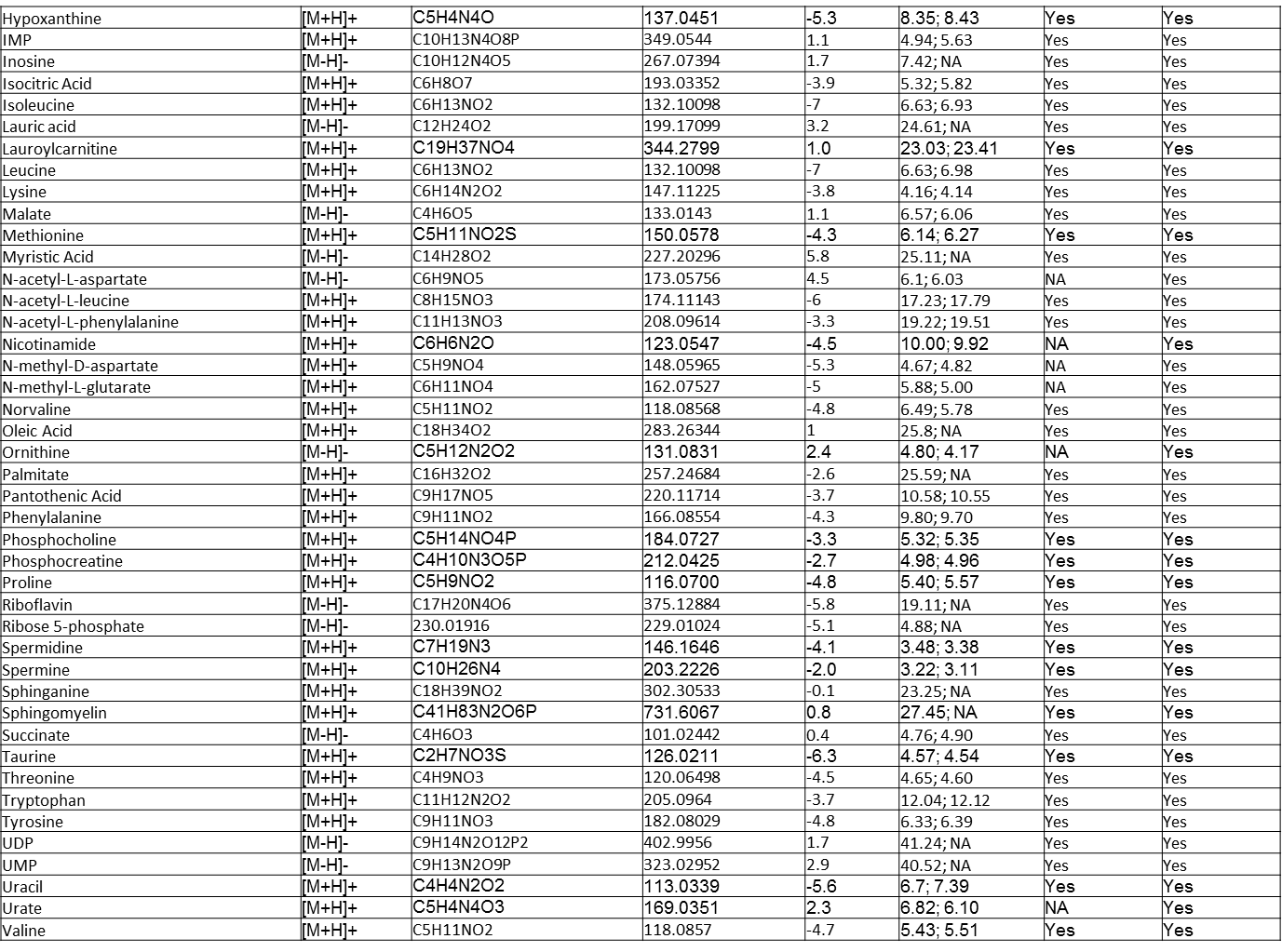
SUPPLEMENTARY TABLE 1: continued.


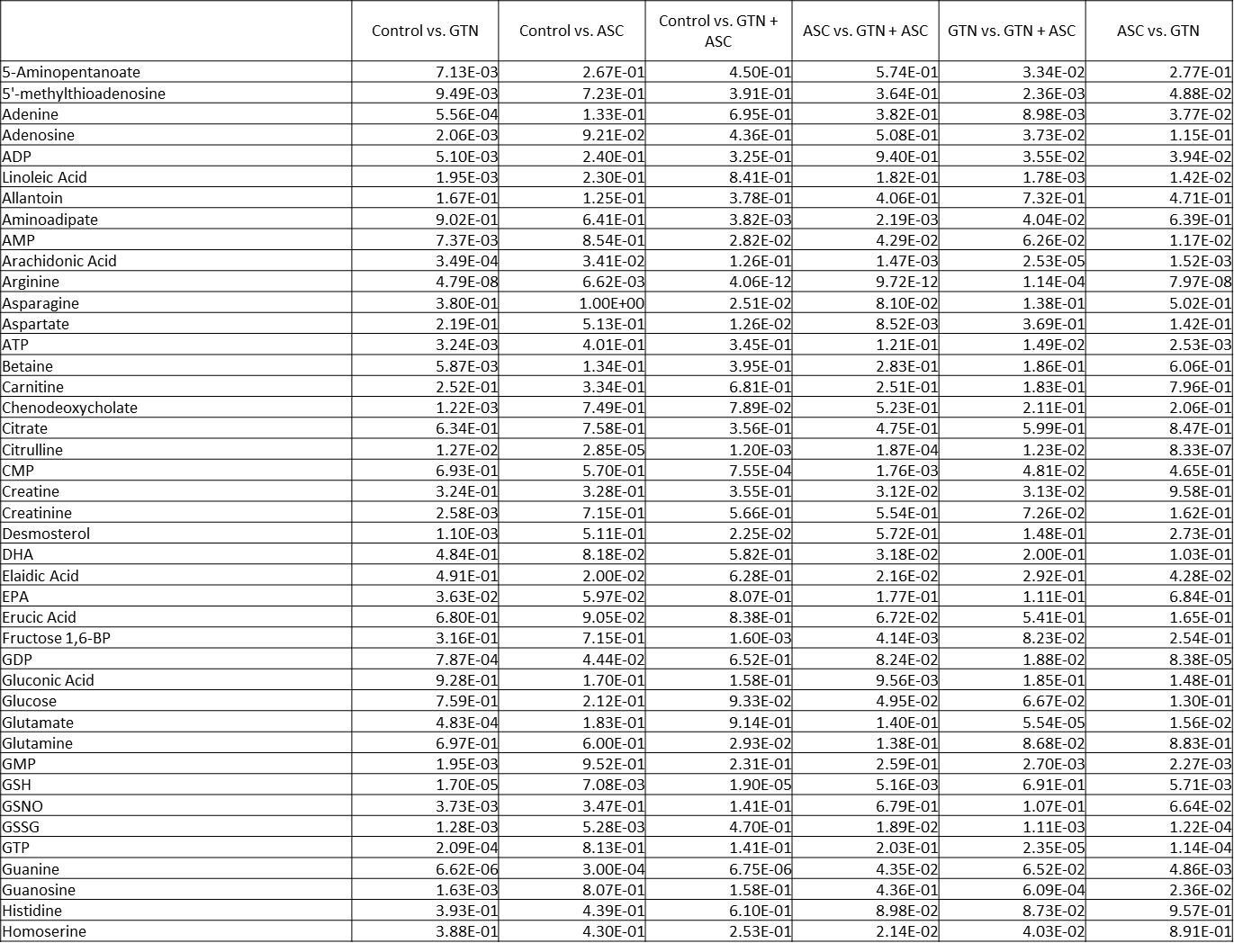
SUPPLEMENTARY TABLE 2. Statistical analysis of metabolite abundances in LLC-PK1 cells. P-values for 85 annotated metabolites were determined by a one-way ANOVA with Fisher’s post-hoc analysis (n=6/group). Holm’s FDR-correction was performed to account for the false discovery rate. FDR-corrected p-values are listed for all groups comparisons.


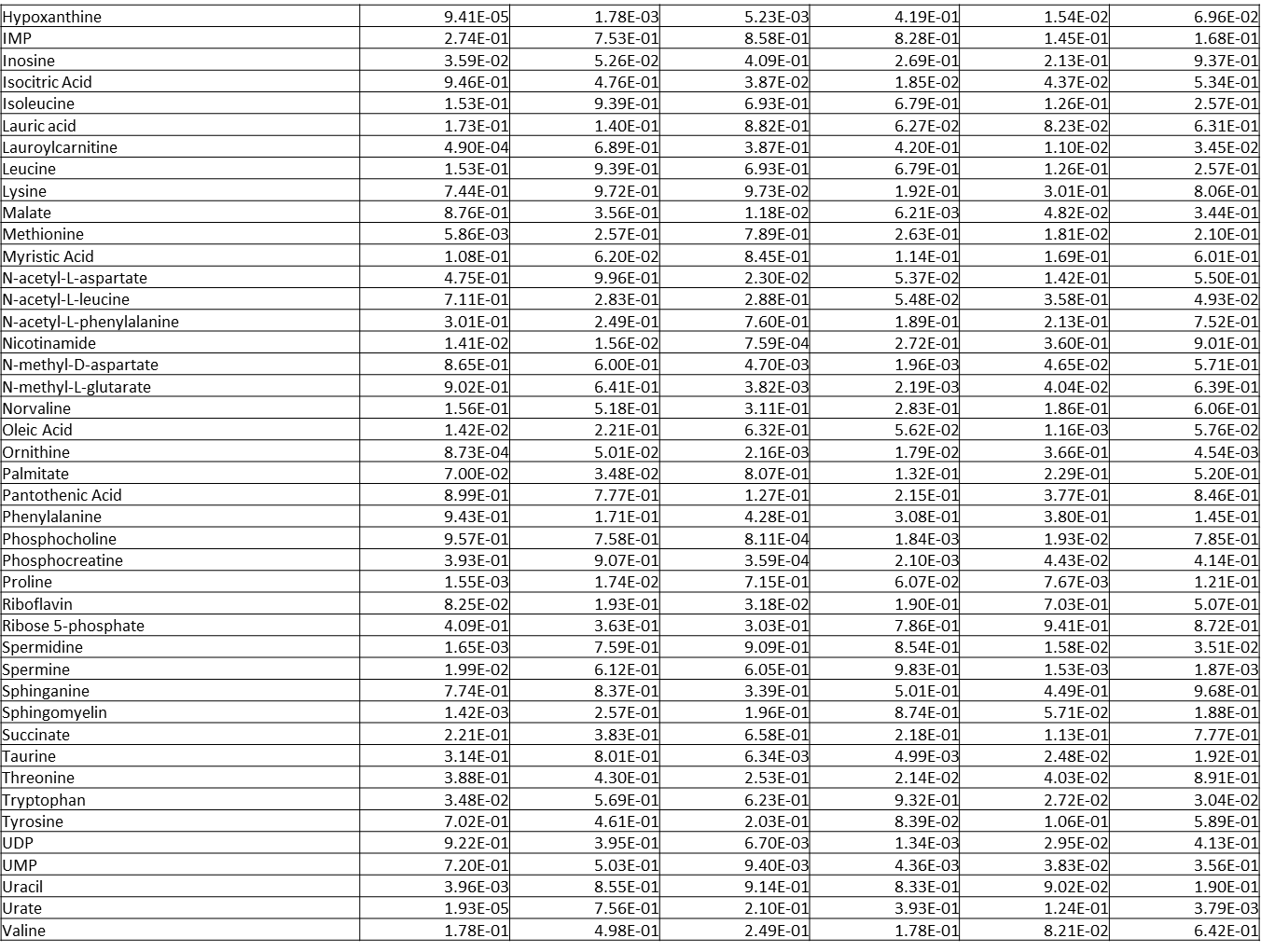
SUPPLEMENTARY TABLE 2: continued.


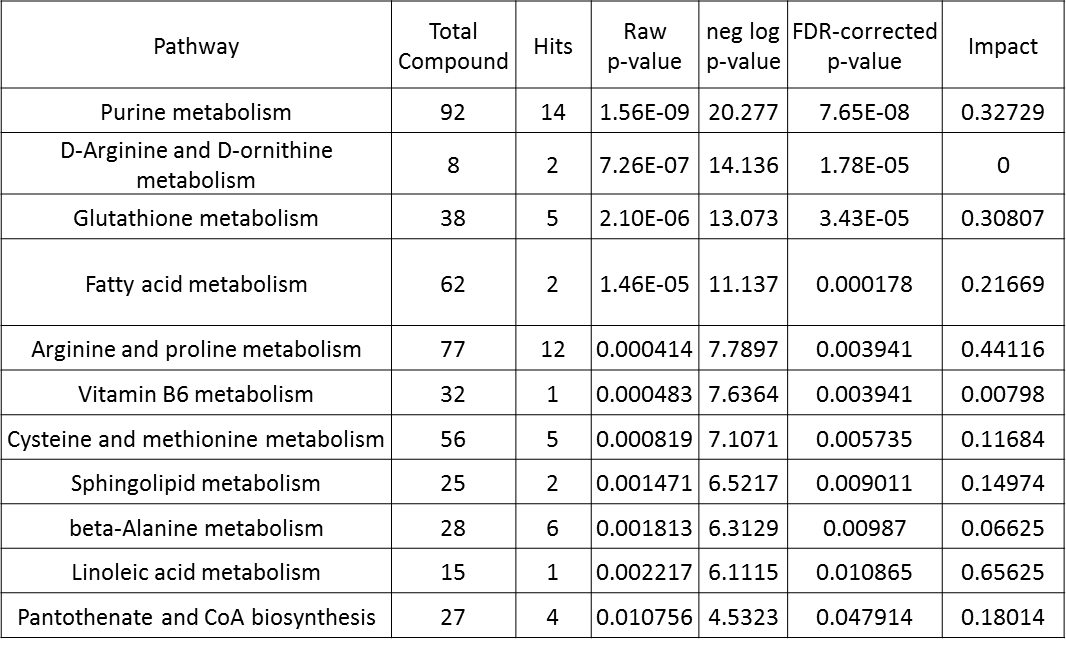
SUPPLEMENTARY TABLE 3. MetaboAnalyst Pathway Analysis. 11 pathways were significantly changed (FDR-corrected p-value < 0.05, n=6/group) between control and GTN-treated cells (MetaboAnalyst 3.0, Pathway Analysis).
